# Trends in greenhouse gas emissions from volatile anaesthetics in 41 countries: 2013–2023

**DOI:** 10.1111/anae.16709

**Published:** 2025-08-06

**Authors:** Marta Caviglia, Andrealuna Ucciero, Andrea Conti, Aurora Di Filippo, Francesco Trotta, Luca Ragazzoni, Francesco Della Corte, Francesco Barone‐Adesi

**Affiliations:** ^1^ Centre for Research and Training in Disaster Medicine, Humanitarian Aid and Global Health Università del Piemonte Orientale Novara Italy; ^2^ Department of Translational Medicine Università del Piemonte Orientale Novara Italy; ^3^ Hospital Pharmacy AOU Maggiore della Carità Novara Italy; ^4^ Italian Medicines Agency Rome Italy; ^5^ Department for Sustainable Development and Ecological Transition Università del Piemonte Orientale Vercelli Italy

**Keywords:** anaesthesia, environment, greenhouse gases, halogenates, volatile anaesthetics

## Abstract

**Introduction:**

Inhalational anaesthetics contribute to greenhouse gas emissions, leading to regulatory restrictions in some countries. This study analysed time trends of greenhouse gas emissions directly attributable to the use of volatile anaesthetic agents in 41 countries.

**Methods:**

Sales data were obtained using data from IQVIA MIDAS**®** and national medicines agencies. We calculated the kilograms of carbon dioxide equivalents (based on global warming potential) per capita and percentage change in greenhouse gas emissions, from the emission of volatile anaesthetics from 2018 to 2023.

**Results:**

Data were obtained for 41 countries, representing approximately 35% of the global population. Greenhouse gas emissions associated with volatile anaesthetic agents decreased in the 27 European Union nations and other ‘western’ countries included in the study (Australia, Canada, New Zealand, UK and USA), achieving in some cases below 0.5 kg of carbon dioxide equivalents per inhabitant. In contrast, several Asian countries showed a substantial increase in emissions, with South Korea and Japan reporting the highest values globally (approximately 2.5 kg of carbon dioxide equivalents per inhabitant). A secondary analysis restricted to European countries showed a 17‐fold difference in per‐capita carbon‐equivalent emissions between the highest and lowest emitters, suggesting that recommendations on the use of volatile anaesthetic agents are implemented inconsistently.

**Discussion:**

Our study highlighted large differences in the management of greenhouse gas emissions attributable to volatile anaesthetic use. While results show a decreasing trend in western countries, albeit with substantial variation, rising trends observed in many Asian countries may constitute a source of concern. The experience of nations that have phased out the highest impacting volatile anaesthetic agents show that reducing emissions below 0.5 kg of carbon dioxide equivalents per inhabitant is attainable. This should serve as a model for other systems, prompting implementation of educational initiatives and specific policies.

## Plain Language Summary

Some gases used to put people to sleep during surgery can harm the environment. Because of this, some countries have made rules to limit their use. This study looked at how these gases have been used over time in 41 countries and how much they added to air pollution from 2018 to 2023. We looked at sales data from companies and national medicine agencies to find out how much of these gases were used. Then we worked out how much pollution (in carbon dioxide equivalents) this created for each person in a country. We also looked at how these numbers changed over time. We got data for 41 countries, which together make up about one‐third of the world's population. In the European Union and other Western countries like Australia, Canada, New Zealand, the UK and the USA, pollution from these gases went down. In some of these countries, it dropped to less than 0.5 kilograms of carbon dioxide per person. But in some Asian countries, like South Korea and Japan, the pollution went up, reaching around 2.5 kilograms per person. In Europe, some countries produced much more pollution than others—up to 17 times more—showing that the rules and practices are not the same everywhere. This study found big differences between countries in how they manage pollution from sleep gases used in surgery. Western countries are mostly lowering their emissions, but in some Asian countries, pollution is going up. Countries that have stopped using the worst gases show that it's possible to reduce pollution to very low levels. Other countries could learn from this and start programmes and rules to help protect the environment.

## Introduction

In recent decades, numerous calls have been made to reduce the climate impact of health systems, which have been calculated to contribute approximately to 5% of global greenhouse gas emissions [1]. With over 300 million patients undergoing general anaesthesia every year [2], anaesthesia practices impact greenhouse gas emissions directly, particularly because of the use and environmental emission of inhalational anaesthetics, and indirectly, for example through energy use, supply chain activities and waste management [3]. This underscores the urgent need for the anaesthetic community to work towards more environmentally friendly clinical practices.

Particularly in Europe, professional societies of anaesthesia have recognised the contribution of their members to the climate crisis, resulting in statements and recommendations [4, 5]. There has been growing concern about the use of desflurane, a halogenated anaesthetic agent that is metabolised minimally by the human body and almost entirely released into the atmosphere [6]. This agent has a global warming potential over 100 years (GWP_100_) of 2540. This metric quantifies the relative impact of a gas on climate change in comparison to an equal mass of carbon dioxide over a 100‐year timescale [6]. While there is an ongoing discussion on whether using global warming potential is the best way for assessing the climate impact of volatile anaesthetic agents, in particular because of their shorter atmospheric lifetime compared with other gases such as carbon dioxide, this approach is currently largely accepted at the international level [7, 8, 9, 10].

Even if desflurane has some advantages in comparison with alternatives due to its rapid onset and offset properties, these do not translate into improved clinical outcomes. Specifically, desflurane has minimal impact on recovery times in the post‐anaesthesia care unit (PACU), postoperative nausea and vomiting (PONV), pulmonary complications and pain scores, raising questions about the justification for its use over suitable alternatives [11]. For these reasons, recent guidelines from the European Society of Intensive Care Medicine recommend the continuous infusion of propofol for maintenance of general anaesthesia and advise that the use of desflurane should be limited to the few cases where it provides clear benefits to the patient [4]. It should also be considered that, despite the advancement of volatile capture technology to absorb volatile anaesthetic agents from scavenging systems, discontinuing the use of desflurane still seems to be the most effective solution to reduce the associated greenhouse gas emissions [12]. Indeed, studies indicate that reducing the use of desflurane in favour of alternative agents (such as sevoflurane, which has a 20‐times lower global warming potential) or total intravenous anaesthesia (TIVA) has the potential to reduce the carbon footprint associated with anaesthetic practice substantially [13, 14]. For these reasons, over the past 2 years Scotland, England and Ireland have announced the decommissioning of desflurane, and the European Union also plans to restrict its use from 2026 onwards [15].

While the issue of greenhouse gas emissions associated with the use of volatile anaesthetic agents is well recognised, national‐level data on how trends have evolved over time are scarce [14]. Indeed, most of the published studies on the climate impact of healthcare systems rely on environmentally extended economic input–output models, which do not consider the actual procurement and usage of anaesthetic gases in their calculations [16]. This information would be useful to shape future policies to reduce the carbon footprint of anaesthesia practices. To fill this knowledge gap, we conducted a multi‐country study to provide a detailed estimation of the greenhouse gas emissions directly attributable to the emissions of volatile anaesthetics.

## Methods

We obtained country‐level sales data for the most used types of volatile anaesthetics (desflurane, isoflurane, and sevoflurane) from two different sources. First, we used IQVIA MIDAS**®** (IQVIA Holdings Inc, Durham, NC, USA) annual volume sales data for 34 countries. This is a proprietary pharmaceutical information service which integrates the national audits by IQVIA into a globally consistent view of the pharmaceutical market, and provides estimated product volumes of registered medicines, trends and market share through retail and non‐retail channels. The IQVIA MIDAS is validated periodically through a standardised and evidence‐based quality assurance programme, known as Accuracy and Timeliness Statistics (ACTS), based on information from more than 5000 pharmaceutical companies and affiliates [17], and has been used widely in global studies on medicines utilisation [18, 19, 20]. As volatile anaesthetic agents are used mainly in hospitals, for this study we considered only countries for which reliable data on hospital sales of these anaesthetics were available (n = 34). We also contacted all the European national medicines agencies and other relevant authorities, which provided data on volatile anaesthetic sales for 14 countries. A preliminary cross‐check that we conducted among countries for which data from both sources (i.e. IQVIA MIDAS and national medicine agencies) were available (n = 7) showed a very high concordance (R^2^ = 0.97). When multiple sources were available for a given country, we used the one that covered the longest time period.

For all the considered volatile anaesthetics, we applied the global warming potential values proposed by Sulbaek Andersen et al. to calculate the carbon dioxide equivalent emissions associated with their usage [12]. When data on volatile anaesthetic agent procurement were expressed in volume rather than mass, we applied density coefficients to convert litres to kilograms (desflurane 1.47, isoflurane 1.50, sevoflurane 1.52). We obtained country population data from the United Nations Department of Economic and Social Affairs [21]. Greenhouse gas emissions are expressed as kg of carbon dioxide equivalents (kg CO_2_e) per capita, to facilitate comparison between countries with different population size.

To further facilitate meaningful comparisons across diverse contexts, we conducted three different analyses, where countries were grouped based on geography; shared socio‐economic characteristics; and data availability. The first analysis compared the 27 European Union nations and other ‘western’ countries (which we define as Australia, Canada, New Zealand, UK and USA), which all have historically high greenhouse gas emissions associated with their healthcare systems. The second analysis focused on the Asian countries for which relevant data were available (China, Hong Kong, Japan, Malaysia, Singapore, South Korea, Taiwan and Thailand). Finally, we made a comparison among all European countries (as defined by the WHO classification). In addition to the 27 member states, this included Belarus, Iceland, Montenegro, Norway, the Russian Federation, Serbia and the UK. For all three groups, we presented time trends of greenhouse gas emissions attributable to volatile anaesthetic procurement and the percentage change between 2018 and 2023.

Statistical analysis was performed using Stata (Release 17, StataCorp LLC, College Station, TX, USA) and R (version 4.2.1, R Foundation for Statistical Computing, Vienna, Austria). The analysis was based on aggregated data and contains no patient‐level or facility‐level information. Hence, this study did not require review by the institutional ethics board.

## Results

Our final dataset included data from 41 countries, representing approximately 35% of the global population. Among the 27 European Union nations, emission data were available for all except Cyprus, Greece, Ireland, Luxembourg and the Netherlands, thus encompassing 92% of the European Union population. Emission data spanned the years 2013–2023 in all countries, with exception of Croatia (2017–2023); Iceland (2019–2023); Latvia (2018–2023); Malta (2017–2023); and Montenegro (2013–2022). Online Supporting Information Table S1 reports the available sources for each country.

**Figure 1:**
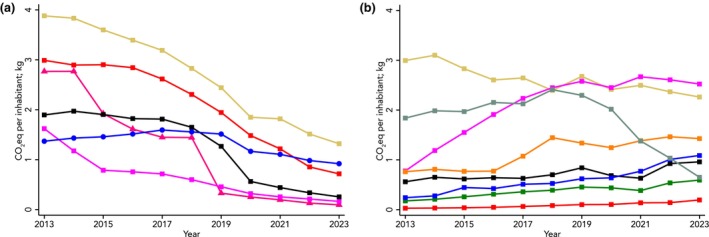
Greenhouse gas emissions trends associated with the use of volatile anaesthetic agents in the 27 European Union nations and western countries and Asian countries. (a) Beige, USA; red, Australia; pink, New Zealand; black, UK; blue, European Union; magenta, Canada. (b) Beige, South Korea; teal, Singapore; magenta, Japan; orange, Taiwan; black, Malaysia; blue, Hong Kong; green, Thailand; red, China. Analysis based on annual volume sales data from IQVIA MIDAS, reflecting estimates of real‐world activity, ©IQVIA, all rights reserved, and National Medicine Agencies (see online Supporting Information Table S1).

**Figure 2:**
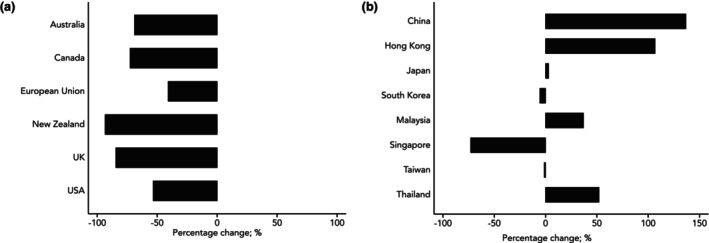
Percentage change in greenhouse gas emissions associated with the use of volatile anaesthetic agents in the 27 European Union nations and western countries (a) and Asian countries (b) from 2018 to 2023. Author analysis based on annual volume sales data from IQVIA MIDAS, reflecting estimates of real‐world activity, ©IQVIA, all rights reserved, and National Medicine Agencies (see online Supporting Information Table S1).

Figures 1 and 2 provide an overview of overall emission trends associated with the use of volatile anaesthetic agents and the percentage change observed in the different countries during the period 2018–2023, respectively.

Data from western countries (Fig. 1a) revealed a clear downward trend throughout the study period. A 65–95% reduction of CO_2_e emissions was observed in Canada, New Zealand and the UK, which achieved levels below 0.5 kg CO_2_e per inhabitant by 2023 (Fig. 2a). On the other side, Australia and the USA continued to exhibit substantially higher levels despite the fact they also cut CO_2_e emissions over time (Figs. 1a and 2a). The 27 European Union nations showed a more complex trend, with an increase in emissions until 2017, followed by a plateau and a reduction from 2020 onward (Fig. 1a).

In contrast with this overall downward trend in western countries, data from Asian countries (Figs. 1b and 2b) showed a substantial increase in emissions associated with the use of volatile anaesthetics. In 2023, South Korea and Japan had the highest per capita emissions globally, approximately 2.5 kg of CO_2_e per inhabitant. Notably, China had a 130% increase in emissions in the period 2018–2023 (Fig. 2b). In absolute terms, it has now become the third largest emitter worldwide associated with the use of volatile anaesthetics (277,882 tonnes of CO_2_e), after the USA (443,312 tonnes of CO_2_e) and the 27 European Union nations (410,726 tonnes of CO_2_e).

**Figure 3:**
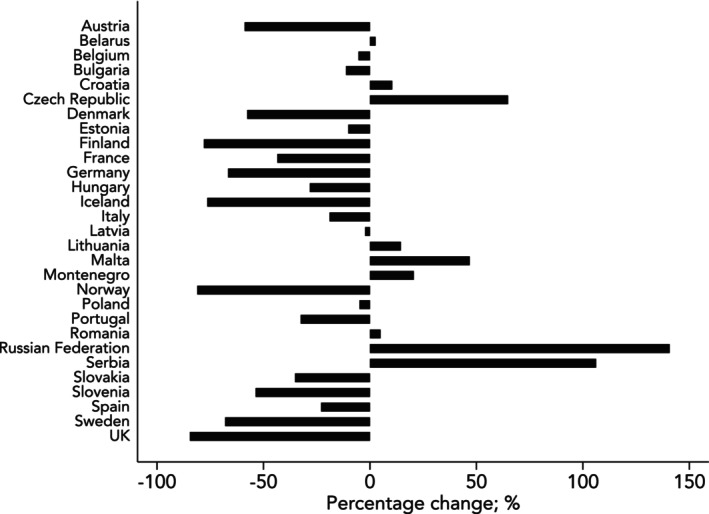
Percentage change in greenhouse gas emissions associated with the use of volatile anaesthetic agents in European Countries (WHO classification) from 2018 to 2023. Author analysis based on annual volume sales data from IQVIA MIDAS, reflecting estimates of real‐world activity, ©IQVIA, all rights reserved, National Medicine Agencies (online Supporting Information Table S1).

In 2018, emission values attributable to volatile anaesthetics in Europe ranged from 2.6 kg CO_2_e per inhabitant (France) to 0.1 kg CO_2_e per inhabitant (Montenegro), reflecting a 43‐fold variation. Many western and southern European countries had emissions well above 1 kg CO_2_e per inhabitant, while eastern European nations reported values below 0.5 kg CO_2_e per inhabitant. By 2023, this heterogeneity has diminished, although it remains substantial, with a 17‐fold variation, from 1.7 kg CO_2_e (Czech Republic) to 0.1 kg CO_2_e per capita in Poland. While many European countries now have emission values below 0.5 kg CO_2_e per capita, Belgium, the Czech Republic, France, Italy and Spain still report values exceeding 1 kg CO_2_e per inhabitant. It is also noteworthy that in some countries, particularly Malta, Serbia, the Czech Republic and the Russian Federation, emissions have increased considerably from 2018 to 2023 (Fig. 3 and online Supporting Information Figure S1).

The analysis of temporal trends among specific European nations highlights distinct patterns across various regions (online Supporting Information Figure S2). In northern Europe, countries such as Finland, Norway and the UK originally had high emissions, ranging from 1 kg to 2 kg of CO_2_e per inhabitant, but then experienced a swift decline, dropping to below 0.5 kg of CO_2_e per inhabitant. In contrast, Denmark, Lithuania and Estonia maintained very low emission levels consistently throughout the study period. Until 2018, Germany reported high emissions (exceeding 2.5 kg CO_2_e per inhabitant) but began a substantial reduction starting in 2019. By 2023, emissions had been reduced to 0.8 kg of CO_2_e per inhabitant. France also achieved significant reductions in emissions from 2018 to 2020, but no further improvements were noted afterwards. Italy, Portugal and Spain have had minimal reductions over time.

## Discussion

Gaining a thorough understanding of the carbon footprint associated with anaesthetic procedures is crucial to inform future policies to reduce greenhouse gas emissions from health systems, aligning with the United Nations Framework Convention on Climate Change (COP‐29) and World Health Organization guidelines [22, 23]. To the best of our knowledge, this study marks the first attempt to provide a detailed, multi‐country estimation of the greenhouse gas emissions directly attributable to the use of volatile anaesthetic agents.

Results reveal a general downward trend of emissions in ‘western’ countries, highlighting a shift toward more sustainable practices. A substantial reduction was observed in Canada, New Zealand, and the UK, potentially driven by awareness campaigns followed by targeted national and subnational policies for the decommissioning of desflurane [5, 13, 15, 24]. The importance of a top‐down approach with policy‐driven interventions has been emphasised in a study conducted in Canadian hospitals, where the authors suggest that the effect of awareness campaigns, education and departmental consensus often plateau without mandates from higher authorities to drive meaningful change [13]. Scotland exemplifies this approach through its National Green Theatres Programme, which evolved from grassroots efforts by clinicians into a government‐supported initiative [25]. The transition away from desflurane was also facilitated by Scotland's national procurement system and the availability of viable substitutes, showing how centralised policies can effectively enable sustainable practices in healthcare [25]. Of note, despite similar efforts in terms of recommendations and decommissioning policies [24, 26], Australia and the USA continue to exhibit higher emission levels than countries with comparable health systems. These results raise questions about the existing barriers, such as potential resistance from healthcare providers; lack of infrastructure to support alternatives; or economic considerations, all of which warrant investigation.

The variability in greenhouse gas emissions associated with the use of volatile anaesthetic agents across European countries further emphasises the influence of national strategies and cultural attitudes toward sustainability. Results obtained in Northern European countries, particularly Scandinavia and the Baltic regions, suggest that achieving volatile anaesthetic‐related emissions below 0.5 kg of CO_2_e per inhabitant is both feasible and sustainable. This success serves as a benchmark for other European countries where reductions remain limited, despite the upcoming European directive to decommission desflurane from 2026 onwards [27]. The consequences of an inconsistent implementation of recommendations are particularly evident in Italy, where previous research found a 25‐fold difference in emissions between regions [28].

We found that the use of volatile anaesthetic agents and the associated greenhouse gas emissions are increasing steadily in several Asian countries. This result is particularly concerning, as it risks offsetting the improvements made in  other countries in last few years. A study conducted in South Korea (which together with Japan had the highest per capita emissions among all the countries considered in our analysis) showed that more than 50% of anaesthetists were unaware of the climate effects of desflurane, but that after training on this topic there was a 50% reduction of desflurane used in the hospitals [29]. This experience underlines that educational interventions can be effective in changing practices in a reasonably short timeframe.

In general, recommendations from various associations and organisations on the reduction of greenhouse gas emissions from halogenated anaesthetic agents converge on three key points: reducing the use of inhaled anaesthetic agents; adopting low fresh gas flow for maintenance of anaesthesia; and prioritising regional anaesthesia or TIVA when possible [4, 26]. However, transitioning to TIVA might present some challenges, including resistance to change of established clinical practices and perceived extra effort, especially among infrequent users [30]. One additional obstacle is the need for additional equipment, such as infusion pumps and extra monitoring to assess depth of anaesthesia [30]. It should also be noted that TIVA is not exempt from its own indirect environmental impacts. Propofol is environmentally persistent and has been shown to be toxic to aquatic life [31]. It is estimated that around 50% of dispensed propofol goes unused and is not always disposed of via appropriate pharmaceutical waste streams [31]. In addition to the pharmaceutical waste, the widespread use of single‐use plastic packaging and administration systems for TIVA (such as ampoules, syringes and infusion lines) contributes significantly to hospital‐generated plastic waste, raising further concerns about environmental sustainability [31]. To this matter, Kanal and Fang [32] argue that the focus should not be on condemning the use of TIVA as an alternative to inhalational anaesthesia, but rather on enhancing its environmental sustainability. For instance, implementing straightforward strategies, such as avoiding the use of excessively large ampoules, reducing unnecessary pre‐preparation of the drug and opting for higher concentrations (2% instead of 1%), could contribute significantly to minimising waste and achieving sustainability objectives [4].

Volatile capture systems have been proposed as a possible solution to prevent the release of volatile anaesthetic agents into the environment [33]. However, clinical studies suggest that the efficiency of these systems is inadequate to prevent an increase in greenhouse gas emissions, particularly from the use of desflurane [33]. Therefore, this approach is currently not considered as a viable alternative to the reduction of volatile anaesthetic agents use, particularly given the uncertain environmental footprint of the entire process and the current regulatory prohibition on reusing recaptured medication [11].

Our study has several limitations. First, in our calculations we considered only the greenhouse gas emissions arising from the atmospheric release of volatile anaesthetic agents. In fact, emissions occur during the whole life‐cycle of a halogenated anaesthetic drug (as for any other pharmaceutical), including the production, packaging, transport, stockage and dismissal stages. However, the vast majority of emissions occur during clinical use due to the direct atmospheric release of unmetabolised gases [3, 16, 34, 35]. Thus, we expect the consequent underestimate in our results to be small. Moreover, it is unlikely that our approach could have substantially biased the time trends that we observed. Second, we were able to include in our analysis only a limited number of countries, with a clear over‐representation of high‐income ones (online Supporting Information Table S1). Indeed, data on sales of volatile anaesthetic agents in South America, Africa and some Asian countries (e.g. India) were either incomplete or unavailable. As desflurane is more expensive than other volatile anaesthetic agents, it is reasonable to expect that most of its use occurs in the (mostly high‐income) countries that we considered. Conversely, we also found that several medium‐income countries (e.g. China and the Russian Federation) increased their use substantially in the last few years. Thus, expanding future research beyond high‐income countries is warranted. Third, is that our analysis was restricted to the use of volatile anaesthetic agents and we did not consider the contribution of nitrous oxide, which is responsible for a substantial proportion of greenhouse gas emissions associated with the use of anaesthetics, and the highest proportion in some systems. This was because reliable consumption data for nitrous oxide are currently unavailable in most countries. Despite its use decreasing over time, nitrous oxide remains readily available in operating theatres worldwide via pipeline systems [36]. Recent studies suggest that direct atmospheric emissions of nitrous oxide are not only a result of operating theatre use but, more critically, come from continuous leaks in the pipeline infrastructure and poor stock management, which lead to a substantial mismatch between procurement and clinical use, and often contribute a much larger volume than that used for clinical purposes [36, 37]. Furthermore, monitoring nitrous oxide use is complicated by its widespread application for analgesia outside of anaesthesia settings, including in labour suites, dental practices and pre‐hospital care environments [38]. Therefore, it is of high priority to improve a thorough quantification of nitrous oxide usage and to implement educational and policy measures aimed at reducing its waste.

Our study has highlighted large differences in the management of greenhouse gas emissions associated with volatile anaesthetic agent use. Overall, results show a decreasing trend in western countries, albeit with substantial variations. In contrast, the rising trends observed in many Asian countries may be a source of concern. The experience of nations that were receptive to awareness campaigns and phased out desflurane use show that reducing emissions below 0.5 kg of CO_2_e per inhabitant is attainable. This success should serve as a model for all the other countries, prompting them to implement educational initiatives for anaesthetists and adopt specific policies to mitigate emissions from inhalational anaesthetics.

## Supporting information


**Figure S1.** Greenhouse gas emissions associated with the use of volatile anaesthetic agents in European countries in 2018 and 2023.
**Figure S2.** Greenhouse gas emissions trends associated with the use of volatile anaesthetic agents in the different European countries.

**Table S1.** Sources of information available for each country and years covered by the data.

Plain Language Summary
