# Supplementary material for: Trends in greenhouse gas emissions from volatile anaesthetics in 41 countries: 2013–2023
Source: Anaesthesia. 2025 Aug 6;80(12):1476–83. doi: 10.1111/anae.16709 (PMC12614409; doi:10.1111/anae.16709)

Figure S1. GHG emissions associated with the use of volatile anaesthetics in European countries (WHO classification) in 2018 (A) and 2023 (B). Results expressed as CO2e per inhabitant, ranging from 0 (blue) to 3 (red). Author analysis based on annual volume sales data from the following sources: (i) IQVIA MIDAS, reflecting estimates of real-world activity. Copyright IQVIA. All rights reserved; and (ii). National Medicine Agencies (see Online Supporting Information Table S1)

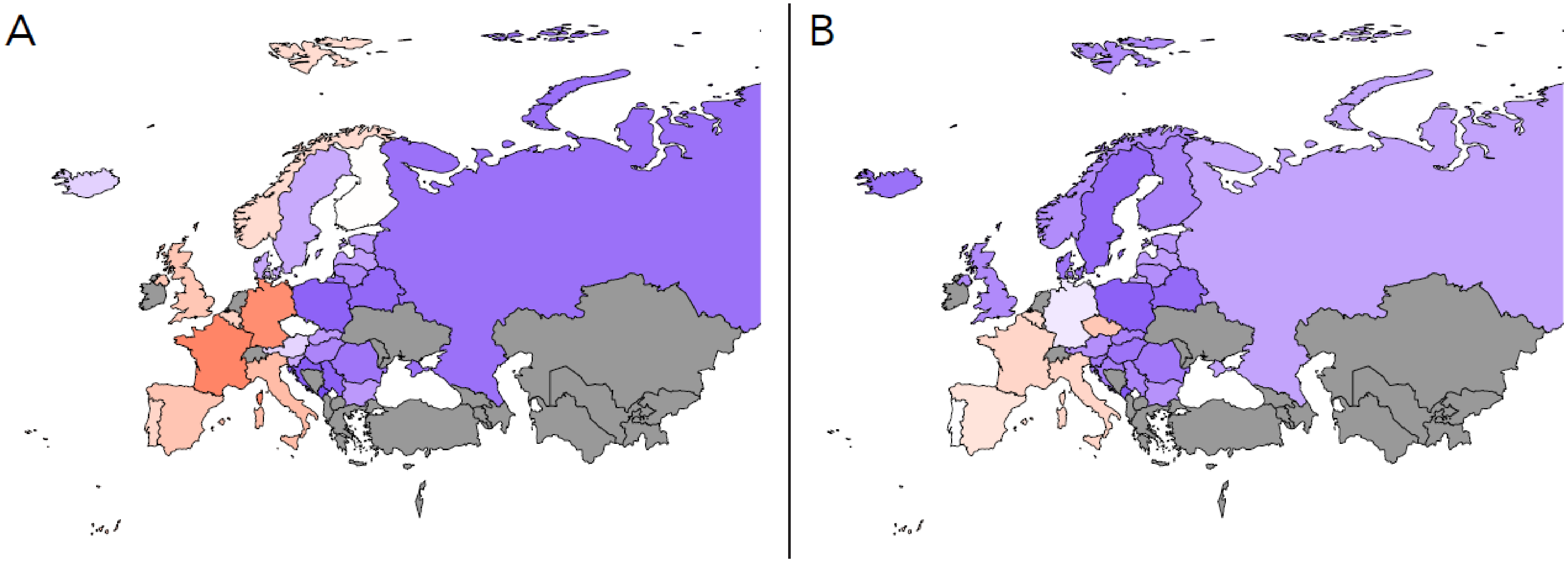

Figure S2. GHG emissions trends associated with the use of volatile anaesthetics in the different European countries. Author analysis based on annual volume sales data from the following sources: (i) IQVIA MIDAS, reflecting estimates of real-world activity. Copyright IQVIA. All rights reserved; and (ii). National Medicine Agencies (see Online Supporting Information Table S1)

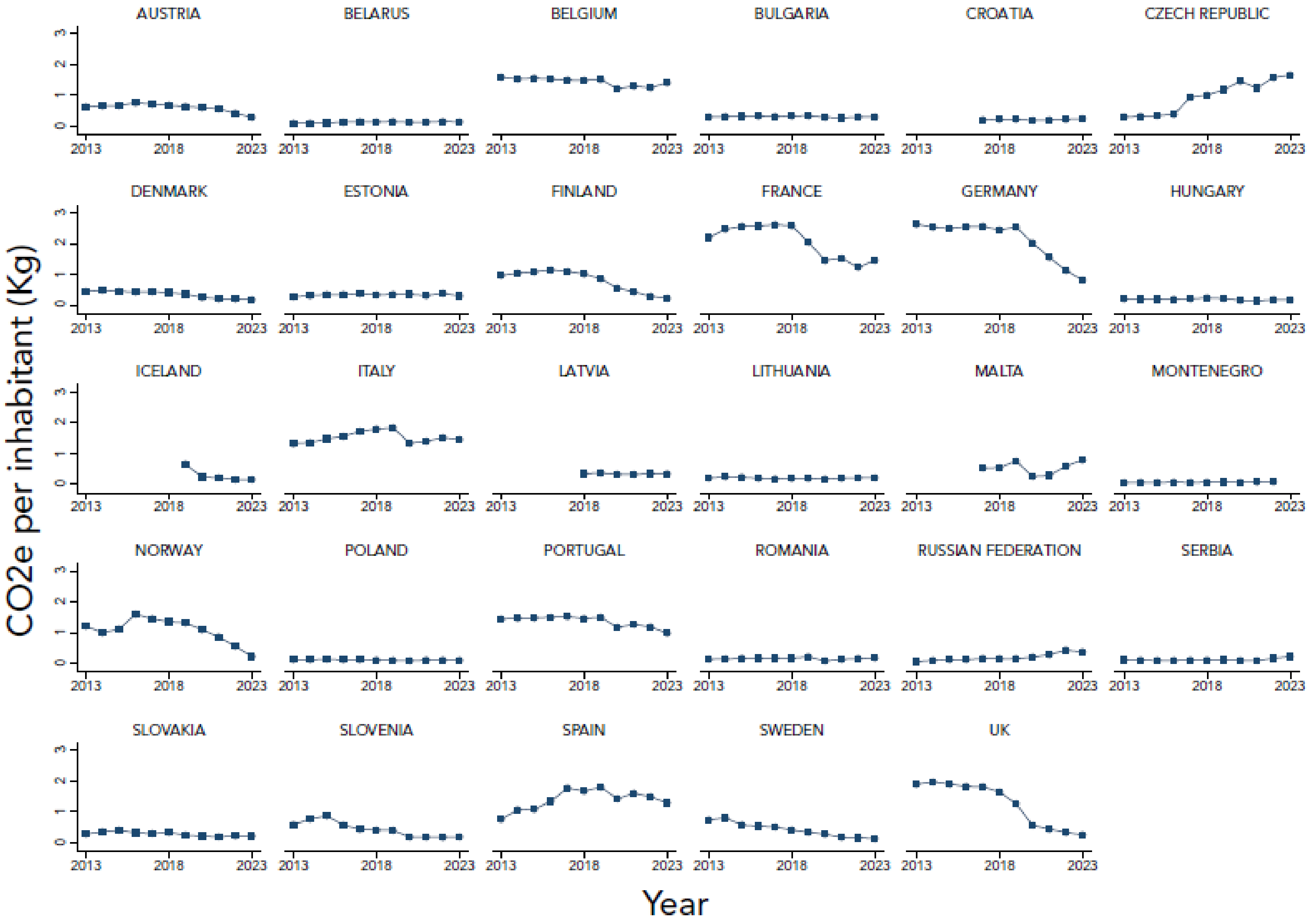

Supplement: Supplementary file 1 — Figure S1. Greenhouse gas emissions associated with the use of volatile anaesthetic agents in European countries in 2018 and 2023. Figure S2. Greenhouse gas emissions trends associated with the use of volatile anaesthetic agents in the different European countries. [file ANAE-80-1476-s002.pdf]
