# Supplementary material for: Trends in greenhouse gas emissions from volatile anaesthetics in 41 countries: 2013–2023
Source: Anaesthesia. 2025 Aug 6;80(12):1476–83. doi: 10.1111/anae.16709 (PMC12614409; doi:10.1111/anae.16709)
Supplement: Supplementary file 2 — Table S1. Sources of information available for each country and years covered by the data. [file ANAE-80-1476-s001.docx]

**Table S1** Sources of information available for each country and years covered by the data. Bold figures indicate which source data has been used for each country. Author analysis based on annual volume sales data from the following sources: (i) IQVIA MIDAS, reflecting estimates of real-world activity. Copyright IQVIA. All rights reserved; and (ii). National Medicine Agencies (see Online Supporting Information Table S1)

| **Country** | **Data from IQVIA (years)** | **Data from National Authorities (years)** | **Source of data** |
| --- | --- | --- | --- |
| Australia | **2013-2023** | NA | NA |
| Austria | **2013-2023** | NA | NA |
| Belarus | **2013-2023** | NA | NA |
| Belgium | **2013-2023** | NA | NA |
| Bulgaria | **2013-2023** | NA | NA |
| Canada | **2013-2023** | NA | NA |
| China | **2013-2023** | NA | NA |
| Croatia | NA | **2017-2023** | Agency for medicinal products and medical devices of Croatia |
| Czech Republic | **2013-2023** | 2019-2023 | State Institute for Drug Control of Czechia |
| Denmark | NA | **2013-2023** | Medstat.dk |
| Estonia | NA | **2013-2023** | Republic of Estonia Agency of Medicines |
| Finland | **2013-2023** | NA | NA |
| France | **2013-2023** | NA | NA |
| Germany | **2013-2023** | NA | NA |
| Honk Hong | **2013-2023** | NA | NA |
| Hungary | **2013-2023** | 2020-2023 | National Public Health and Pharmaceutical Centre |
| Iceland | NA | **2019-2023** | Icelandic Medicines Agency |
| Italy | **2013-2023** | 2013-2023 | Italian Medicine Agency |
| Japan | **2013-2023** | NA | NA |
| Latvia | NA | **2018-2023** | State Agency of Medicines of the Republic of Latvia |
| Lithuania | **2013-2023** | 2014-2023 | State Agency of Medicines of Lithuania |
| Malaysia | **2013-2023** | NA | NA |
| Malta | NA | **2017-2023** | MHA-Central Procurement and Supplies, Ministry for Health and Active ageing |
| Montenegro | NA | **2013-2022** | Institute for Medicines and Medical Devices |
| New Zealand | **2013-2023** | NA | NA |
| Norway | **2013-2023** | 2013-2023 | Norwegian Drug Wholesales Statistics, Norwegian Institute of Public Health |
| Poland | **2013-2023** | NA | NA |
| Portugal | **2013-2023** | 2018-2022 | National Authority of Medicines and Health Products of Portugal |
| Romania | **2013-2023** | NA | NA |
| Russian Federation | **2013-2023** | NA | NA |
| Serbia | **2013-2023** | NA | NA |
| Singapore | **2013-2023** | NA | NA |
| Slovakia | **2013-2023** | NA | NA |
| Slovenia | **2013-2023** | 2019-2022 | National Institute of Public Health of the Republic of Slovenia |
| South Korea | **2013-2023** | NA | NA |
| Spain | **2013-2023** | NA | NA |
| Sweden | **2013-2023** | NA | NA |
| Taiwan | **2013-2023** | NA | NA |
| Thailand | **2013-2023** | NA | NA |
| UK | **2013-2023** | NA | NA |
| USA | **2013-2023** | NA | NA |
