# Supplementary material for: Trends in greenhouse gas emissions from volatile anaesthetics in 41 countries: 2013–2023
Source: Anaesthesia. 2025 Aug 6;80(12):1476–83. doi: 10.1111/anae.16709 (PMC12614409; doi:10.1111/anae.16709)
Supplement: Supplementary file 3 — Plain Language Summary [file ANAE-80-1476-s003.docx]

**Plain Language Summary**
Some gases used to put people to sleep during surgery can harm the environment. Because of this, some countries have made rules to limit their use. This study looked at how these gases have been used over time in 41 countries and how much they added to air pollution from 2018 to 2023. We looked at sales data from companies and national medicine agencies to find out how much of these gases were used. Then we worked out how much pollution (in carbon dioxide equivalents) this created for each person in a country. We also looked at how these numbers changed over time.
We got data for 41 countries, which together make up about one-third of the world’s population. In the European Union and other Western countries like Australia, Canada, New Zealand, the UK and the USA, pollution from these gases went down. In some of these countries, it dropped to less than 0.5 kilograms of carbon dioxide per person. But in some Asian countries, like South Korea and Japan, the pollution went up, reaching around 2.5 kilograms per person. In Europe, some countries produced much more pollution than others—up to 17 times more—showing that the rules and practices are not the same everywhere. This study found big differences between countries in how they manage pollution from sleep gases used in surgery. Western countries are mostly lowering their emissions, but in some Asian countries, pollution is going up. Countries that have stopped using the worst gases show that it's possible to reduce pollution to very low levels. Other countries could learn from this and start programmes and rules to help protect the environment**.**
